# Wearables as Translational Physiomarkers and Clinical Endpoints in Insomnia Research: Can Sleep Research Advance Psychiatry?

**DOI:** 10.1111/jsr.70028

**Published:** 2025-02-28

**Authors:** Victor I. Spoormaker, Borbala Blaskovich

**Affiliations:** ^1^ Max Planck Institute of Psychiatry Munich Germany

**Keywords:** actigraphy, insomnia, pulse photoplethysmography

## Abstract

Wearables that integrate actigraphy and pulse photoplethysmography (ACT + PPG) could represent a promising advancement in insomnia research and clinical practice. This especially applies to assessing objective sleep for a longer period in the home environment, which is impractical with ambulatory polysomnography (PSG) whereas actigraphy alone struggles with detecting wake‐after‐sleep‐onset, as one of the most important variables for insomnia research (on which further variables, such as sleep efficiency, depend). The addition of heart rate and heart rate variability data to actigraphy strongly enhances WASO detection, offering hope for objective WASO detection for insomnia. For further physiomarker development, several challenges of ACT + PPG need to be tackled. Current commercial solutions often rely on “black‐box” algorithms trained on small, healthy samples, leading to inaccuracies in sleep‐disordered populations. Moreover, the lack of access to raw data hinders validation and cross‐study comparability. These and other issues are addressed in detail in this opinion paper in order to raise awareness and start a discussion about more reliable, objective sleep markers that could be readily used as objective clinical endpoints in clinical trials on cognitive‐behavioural therapy for insomnia (CBT‐I) or stress‐related mental disorders and novel pharmacological compounds. The sleep research community has the opportunity to establish ACT + PPG as a gold standard of home based, longitudinal sleep monitoring, which has the potential to bridge the gap between research and clinical practice, transforming clinical trials and improving psychiatric care.

## 1 Objective Sleep Detection in the Home‐Environment: Polysomnography, Actigraphy and the Problem of Wake‐After‐Sleep‐Onset (WASO)

For assessing objective sleep, polysomnography and actigraphy are two of the most commonly applied methods, neither of which has utility for diagnosing insomnia. Anyone who has ever slept one or two nights in a sleep laboratory with polysomnography knows that this is not the best way to estimate the amount of natural sleep (Bruyneel et al. 2011; Montserrat Sánchez‐Ortuño et al. 2010), sleep onset latency and wake‐after‐sleep‐onset (WASO) in the home environment, so it is hardly surprising that studies employing polysomnography have had difficulties in the past to find large and robust differences in sleep variables between individuals with insomnia and healthy controls (Baglioni et al. 2014; Zhao et al. 2021). Measuring polysomnography in the home‐environment for days if not weeks would be a solution to sleep being affected by the artificial environment of the sleep laboratory, but still there is something unusual (wet electrodes on the head or dry electrodes pressing into the forehead) to which individuals may or may not habituate. We have for instance investigated the use of Dreem 2 EEG headbands (until its discontinuation in the Summer of 2023), for assessing sleep in psychiatric outpatients with mild affective/anxiety symptomatology, and noted that a majority managed to sleep with the headbands for a handful of nights in a row, while providing decent data quality (Blaskovich et al. 2023). However, there were multiple healthy controls and psychiatric outpatients complaining about sleeping with such a headset as well, and 4‐week measurements do not seem feasible, let alone longer periods.

By contrast, actigraphy alone in the home‐environment has high utility for clinical trials for assessing motion and motion‐related variables (including circadian rhythm parameters), just not for assessing sleep in individuals with insomnia. Actigraphy measures wrist activity and misses most of WASO and underestimates sleep‐onset‐latency (SOL; Marino et al. 2013; Montserrat Sánchez‐Ortuño et al. 2010; for example, in one key study, the specificity for sleep (=detection of wakefulness) of actigraphy was 35%; Marino et al. 2013). This problem, which affects downstream sleep parameters such as sleep duration and efficiency, cannot be solved by activity measurements alone, as individuals with insomnia happen to lie still in the bed while awake for quite a bit as this is their core sleep problem. No fitting of parameters in complex non‐linear models will change the fact that the relevant information on SOL and WASO is not acquired with motion sensors alone (Rösler et al. 2023).

Since 2016, first smartwatches and since last year also research‐grade actigraphs (e.g., the LEAP by actigraph.com) have added pulse plethysmography (PPG) to actigraphy, which could be a game‐changer for WASO detection. With other devices and heart‐rate signal, for example, electrocardiography, it was already clear that adding heart rate (HR) and heart rate variability (HRV) values improves WASO detection not just in healthy subjects but also in insomnia patients (Fonseca et al. 2020; Wulterkens et al. 2021). This jump in accuracy for detecting WASO is important for future clinical trials as it overcomes the major limitation of actigraphy for insomnia research. The technological readiness of this ACT + PPG approach is decent, and it could already be used in open label trials by savvy clinical researchers.

## 2 Arguments Against Current Wearable and Analogue Solutions

**FIGURE 1:**
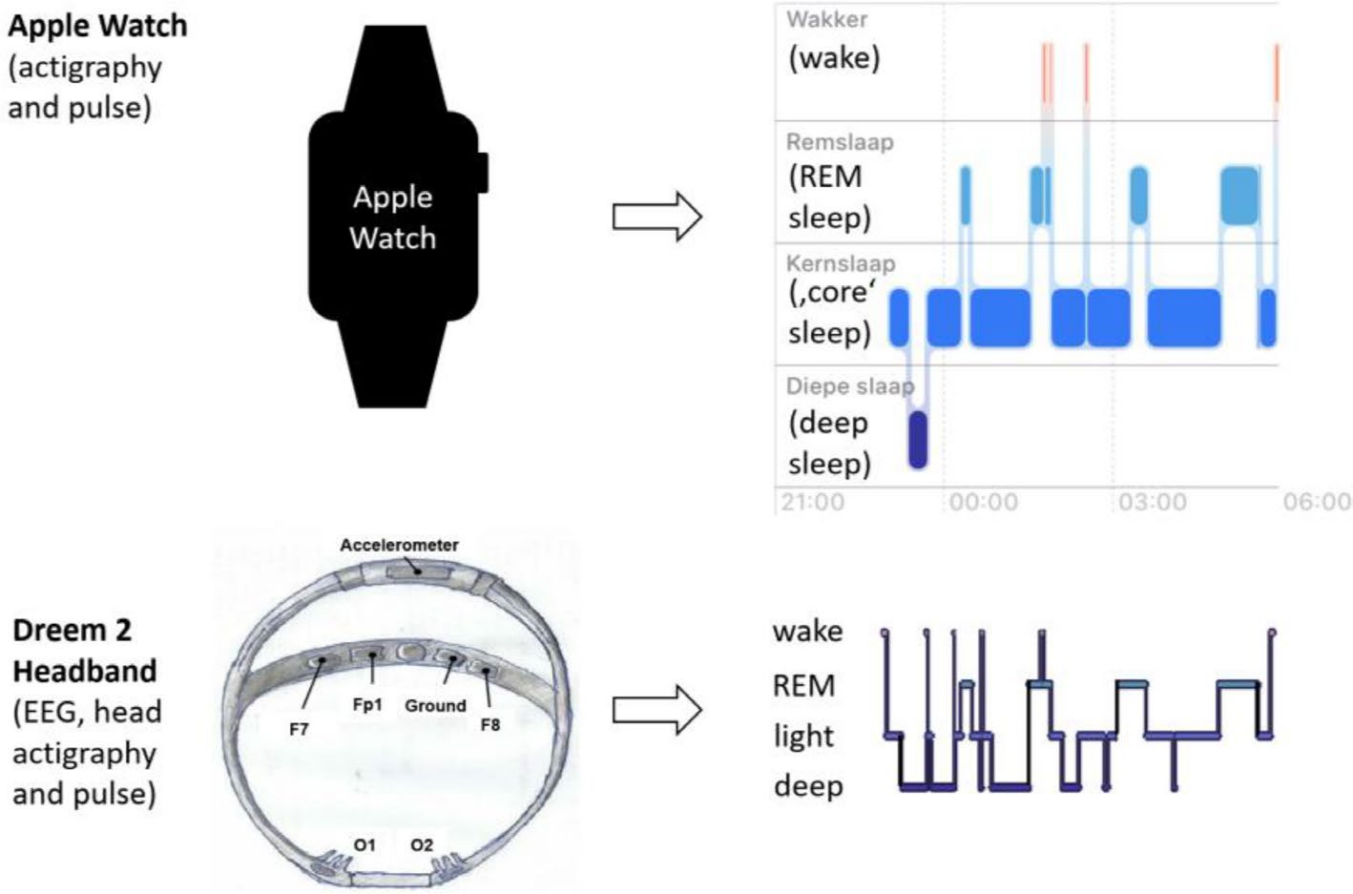
Decent wake‐after‐sleep‐onset detection, misclassifying deep sleep: ACT + PPG in a nutshell. *Source*: Upper right panel generated in Apple Health (iOS 18). Lower left panel reprinted with kind permission of Hannah Neumayer (Bullón Tarrasó 2022).

So why don't we? Current problems with commercial ACT + PPG solutions in smartwatches or fitness‐trackers are at that they are based on black‐box algorithms of commercial wearable producers, on *ever‐changing* black‐box algorithms of commercial wearable producers at that, an overfitting of deep learning algorithms for sleep staging of sleep of healthy subjects of small samples in California, and that researchers have no access to raw ACT + PPG data at the required resolution but get summary values instead, if at all (de Zambotti et al. 2024). This situation is clearly not helped by providing adventurous 4‐class classifications in commercial app solutions (instead of 2‐class, sleep‐versus‐wake classifications, which goes relatively well with ACT + PPG, the attempt is to classify deep sleep, rapid eye movement [REM] sleep, light sleep and wakefulness). This appears to result in massive deep sleep underestimations (Fonseca et al. 2020) in several individuals (e.g., around half an hour per night for months, see Figure 1 for a representative illustration). As a result, there is a healthy scepticism among sleep researchers against using wearable technology as clinical endpoints for individuals with insomnia. However, commercial wearables typically also provide two‐class sleep‐versus‐wake classification, which appears generally more robust, as long as the software version remains the same. Research‐grade ACT + PPG devices providing raw data access, and ongoing validations of commercial ACT + PPG devices and their algorithms against mobile EEG solutions will help take away such doubts, but there is some way to go still (Menghini et al. 2021; de Zambotti et al. 2022).

A more difficult issue is the existence of easy‐to‐use sleep diaries and smartphone‐apps with subjective ratings. Compared to passive ACT + PPG solutions, diaries are much less informative and reliable when it comes to sleep and WASO estimations (Lehrer et al. 2022). The main issue is a logical flaw, as in CBT‐I, individuals are typically instructed not to look at the clock in the night, as to prevent counting the minutes and hours, but should in the morning report from when to when they were awake, while not having looked at the clock. For CBT‐I trials, the sleep diary used to be the only viable option, but this is no longer the case. Moreover, the argument that sleep wearables lead to obsessing with sleep duration, which is certainly a problem for individuals with insomnia, seems to equally apply to sleep diaries. For ACT + PPG wearables, the feedback can even be switched off or changed (e.g., showing sleep efficiency instead of sleep duration). Sleep diaries still have their merit, particularly for assessing subjective sleep quality and sleep impressions, and objective and subjective measurements could be readily combined to better understand efficacy of newer therapy forms such as digital CBT‐I (e.g., Maurer et al. 2024).

A hard problem is the argument that we have come across repeatedly, namely that if one views insomnia as a purely subjective problem (i.e., there is not much wrong with actual sleep but it is‐more‐a‐sort‐of‐sleep‐state‐misperception) objective sleep is irrelevant. The current subjective clinical endpoints in insomnia trials, with subjective insomnia severity and subjective sleep diaries, happen to fit the current CBT‐I focus rather well. By contrast, objective markers do not seem to add much to such a purely subjective approach.

We would like to argue that this need not be the case and that all clinicians should want to be informed about the actual sleep of the people they are trying to help. There are a few reasons for this: First, the clinical diagnoses of insomnia still incorporate difficulties falling asleep or sleeping through on a given amount of nights per week (American Academy of Sleep Medicine 2023; American Psychiatric Association 2013; World Health Organisation 2018), so it would be useful to actually know if that is the case. As with other disorders and diseases in sleep medicine and other medical fields, objective test results could similarly guide diagnosis and treatment (imagine a cardiologist not using a portable electrocardiographic device but exclusively relying on self‐reported symptoms for diagnostic purposes).

Second, tracking objective sleep has the benefit of allowing treatment adherence monitoring, for instance for sleep restriction, important for examining and improving treatments (Spina et al. 2023).

**FIGURE 2:**
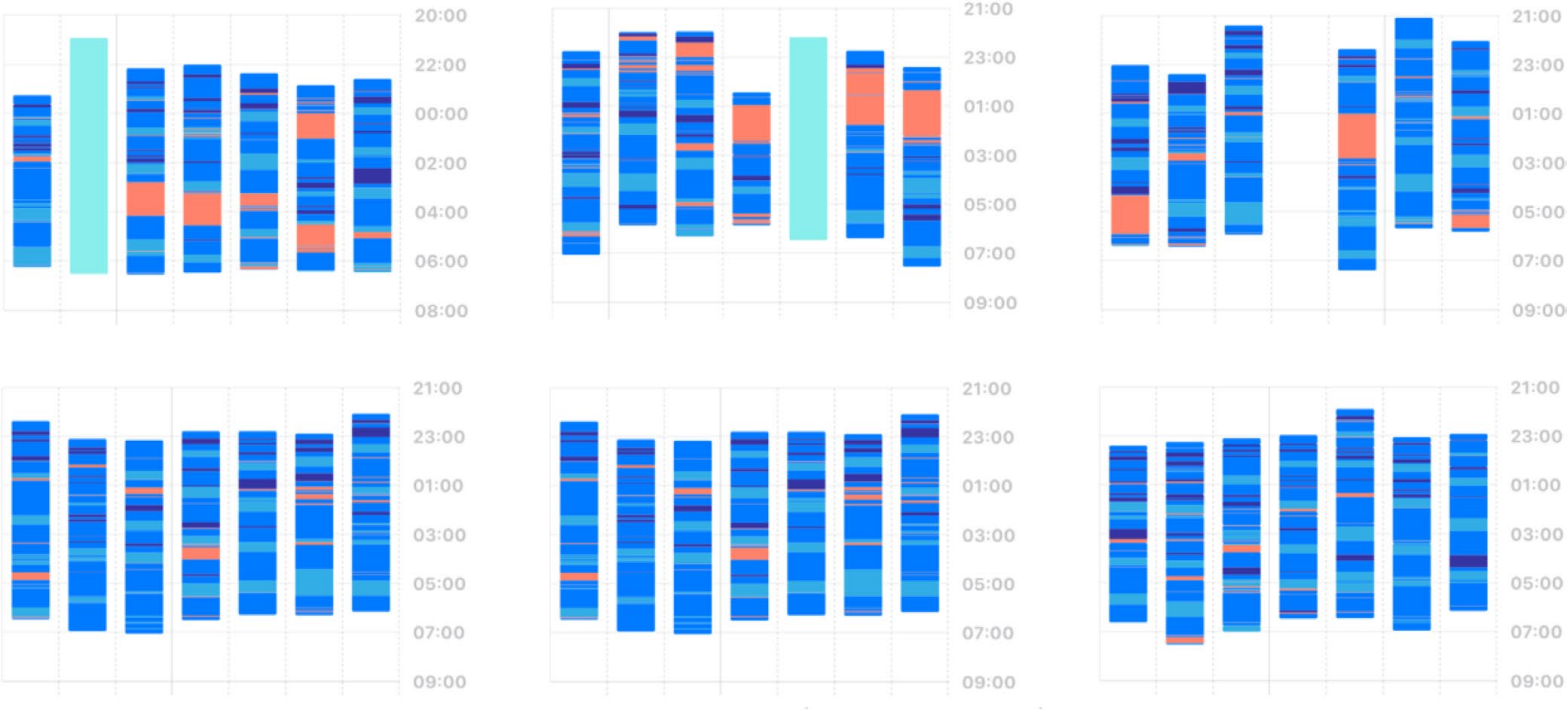
Illustration of long‐term sleep and WASO measurements in the home‐environment: Sleep patterns of a situational insomniac (high sleep reactivity). Blue colour marks sleep (shading should be ignored) and orange awake periods. The three upper panels reflect weeks with high perceived stress, the three lower panels with low perceived stress. *Source*: Plots generated in Apple Health (iOS 18).

**FIGURE 3:**
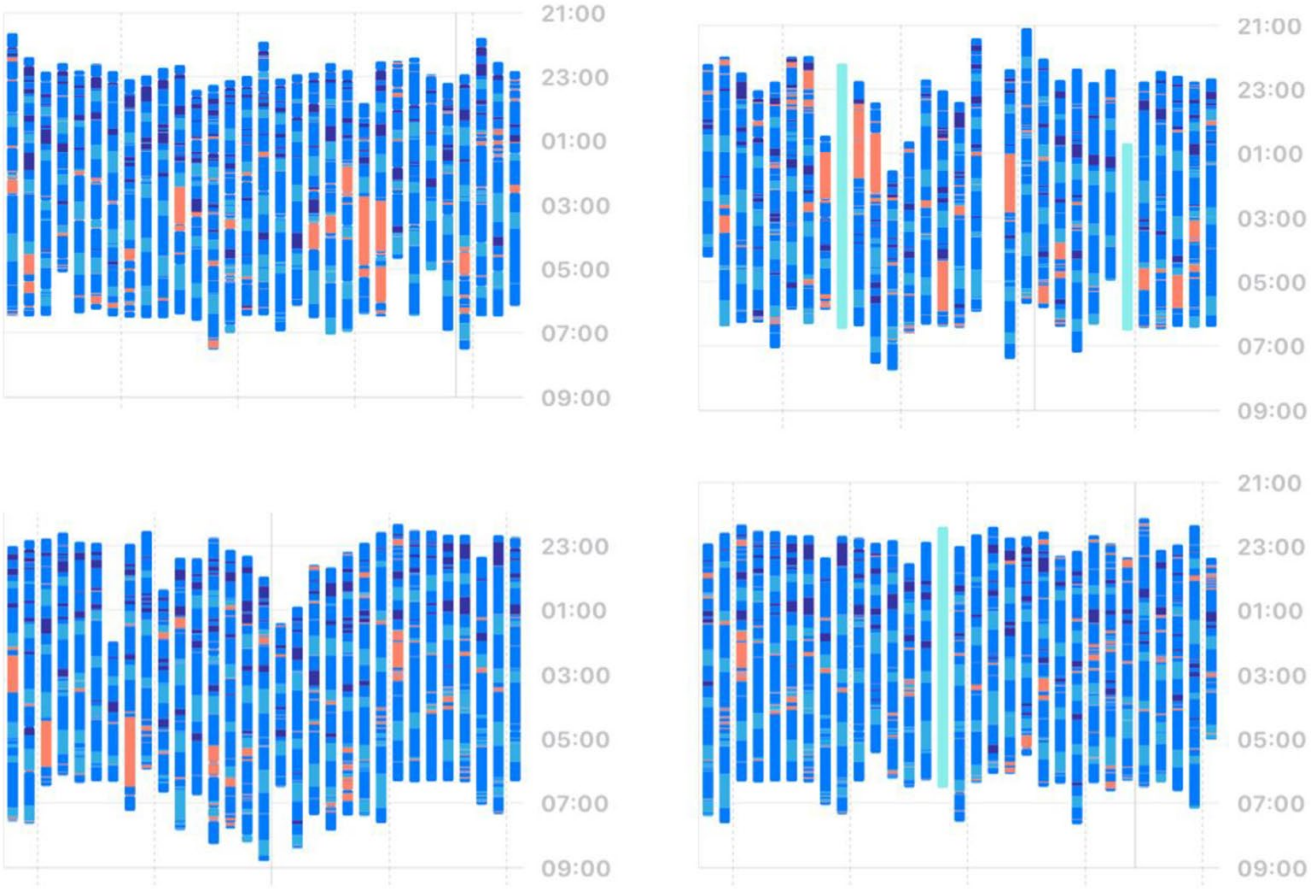
Illustration of long‐term sleep and WASO measurements in the home‐environment: Upper left panel represents issues sleeping through; upper right panel contains disruptions immediately after falling asleep; the lower left panel shows circadian variability during the holidays; the lower right panel depicts a relatively ‘good’ month of sleep. *Source*: Plots generated in Apple Health (iOS 18).

Third, tracking actual sleep will allow us to better stratify individuals with insomnia into subtypes (Ferini‐Strambi et al. 2019; Miller et al. 2016). Some subtypes have already been proposed, like the subtype of insomnia with objective short sleep duration (e.g., sleep efficiency < 85%, sleep amount < 6 h), which appears a more severe subtype of the disorder related to risk of hypertension in a recent meta‐analysis (Dai et al. 2024; Meng et al. 2013). In the other direction, the case of situational, stress‐related insomnia or sleep reactivity comes to mind (Drake et al. 2004; Kalmbach et al. 2018), which is typically assessed with a questionnaire but could as well be tracked with ACT + PPG devices, see Figures 2 and 3 for an illustration. After all, some individuals are highly sleep reactive to stress, will have a longer SOL or more WASO, and we can track this over months. Although this subtype appears more relevant for stress‐related research, it could be relevant for understanding prodromal phases of chronic insomnia (Reffi et al. 2023; Yoo et al. 2023).

Finally, measuring objective sleep over months in the home‐environment could in the end be more causally informative, as sleep fragmentation can be quantified, circadian rhythms disentangled and stressful periods analysed. Night‐to‐night variability is so far an underestimated and underrecognized measure, due to the mostly one night lab based nature of sleep diagnostics (Chouraki et al. 2023). However, due to wearable devices its importance is being slowly recognised. Multi‐night measurements could be especially informative in case of sensitive groups such as elderly, insomniacs or people with nightmare disorders (Buysse et al. 2010; Ghazi et al. 2025; Paquet et al. 2024; Zheng et al. 2024) where increased night‐to‐night variability of SOL, WASO and TST could be informative markers. Furthermore, multiple nights of measurement seems to be also important in the identification of sleep related breathing issues such as OSA (Roeder et al. 2021) which are highly prevalent (35%–54%) in insomnia (Sweetman et al. 2019).

The clinical use of available, objective data would also benefit clinical research and particularly the methodological issue of psychotherapeutical treatment trials of fully relying on subjective reports after a non‐blinded intervention. Patients know they received an intervention, and afterwards subjectively evaluate to what extent they think all this work from themselves and the researchers/clinicians has helped them. This may be (or have been) the standard in clinical CBT research, but clinical pharmacological studies in sleep medicine and psychiatry use either double‐blinding in a placebo‐controlled trial, objective endpoints, or both, thus mitigating this problem.

## 3 Insomnia Research Should Not Strive to Be Like Depression and Anxiety Research, But Rather Advance It

A last argument is that insomnia should stick to subjective readouts akin to how depression and anxiety disorders are handled in research and practice. But that there are no biomarkers yet for depression is a tragedy not an aspiration, as it impairs our understanding of this collection of rather arbitrarily combined symptoms and medication effects. For instance, even though selective serotonin reuptake inhibitors (SSRIs) have decent effects on core depressive symptoms such as depressed mood and anhedonia (Eriksson and Hieronymus 2018; Hieronymus, Emilsson, et al. 2016a; Hieronymus, Nilsson, et al. 2016b), they also tend to worsen sleep as a side‐effect (Hutka et al. 2021). In a world with biomarkers, the effect of SSRIs on a depressed mood marker or objective test for anhedonia would not be questioned, and the side‐effect of disrupted sleep would be objectively monitored to get a treatment effect/side‐effect profile. With our current subjective assessments, the improvements on core depressive symptoms are cancelled out by worsened scores on sleep items on the same rating scale, and bona fide effects are masked and casted in doubt. Therefore, instead of aiming to be like psychiatry, insomnia research should rather strive to bring psychiatry forward and provide usable, scalable digital biomarkers for sleep. These physiomarkers have the potential to be easily embedded into everyday clinical practice and not just transform research on insomnia but also other stress‐related psychiatric disorders (e.g., depression, posttraumatic stress disorder). Next to providing more reliable individual data on actual sleep, these above mentioned wearables allow the collection of unseen large‐scale datasets that can be used to validate clinical endpoints (de Zambotti et al. 2024; Zheng et al. 2024).

This could also help us understand the topic of treatment‐resistance better. For instance in depression, it is estimated that only around 30%–40% of patients achieve full remission after first‐line pharmacological treatment (Gloster et al. 2020; Rush et al. 2006). Non‐responders enter a conceivably long phase of trial‐and‐error with other medications, the effects of which become noticeable after a couple of weeks (Kudlow et al. 2014), potentially ending with inpatient treatment or electro‐convulsive therapy (Subramanian et al. 2022). The absence of objective measurements and biomarkers makes it challenging to determine which patients should receive SSRIs, SNRIs, other pharmacological treatments, or psychotherapy alone. Clinical insomnia research could also address the topic of treatment‐resistance in more detail and extend it by quantifying treatment‐resistance in an objective manner: which patients actually sleep more after CBT‐I, who gains how much of additional sleep, for whom is the sleep less fragmented or more efficient, or which subgroups do not respond at all and what should we do about it? We see this possibility to objectively quantify the disorder as a primary advantage of clinical insomnia research, since sleep is a physiological state that can be measured objectively.

## 4 Research Agenda

As it was mentioned earlier, the use of ACT + PPG wearables holds transformative potential for sleep related psychiatric research. The validation/performance evaluation and dissemination of ACT + PPG algorithms for two‐class (sleep vs. wakefulness) classifications are highly important for this to work, since currently the majority of labs are using their own “home‐made” tools or receives their already pre‐processed data from various commercial wearable companies with their own not fully validated algorithms (de Zambotti et al. 2024). Even though differences among companies might not be that large, the black‐box nature makes it hard to compare and contrast results across different studies. Just as PSG related sleep research has the AASM guidelines of how to analyse the data, the ACT + PPG wearable research community would also need to agree upon its main analysis ground rules. We should put particular emphasis on using research‐grade wearable devices that can provide raw and high‐resolution data that can be easily coupled with open access algorithms (Python, Matlab or R based to maximise availability). In case of using commercial wearables, access to the raw data is much harder, but then it is essential to use the same software version throughout the full study durations since any update can contain any algorithm update, which inevitably alters data interpretation. Furthermore, one should always keep in mind that these black‐box algorithms are mostly trained on healthy sleeping young individuals which results in increased inaccuracy when it comes to sleep detection in a sleep disordered group (de Zambotti et al. 2024). Collecting ACT + PPG databases with different sleep disorders and training deep learning algorithms per population would be strongly advisable.

This validation/performance evaluation across devices and algorithms would be also crucial to close the gap between research and practical applicability. Objective algorithmic endpoints such as WASO, SOL, number of awakenings etc. could be easily validated against other home‐based measurements like EEG‐headbands, thus improving the precision of the proposed biomarkers. This allows exploring their utility as clinical endpoints in pilot studies for evaluating the efficacy of CBT‐I or novel medications in early drug discovery trials.

## 5 Conclusion

Our hope is that just with a little effort and collaboration from the scientific research field ACT + PPG measurements could become the new gold standard for monitoring sleep, SOL, WASO and sleep efficiency in the home environment. This would be highly relevant for more efficient sleep monitoring in future clinical trials and eventually for day‐to‐day clinical practice.

## Author Contributions

**Victor I. Spoormaker:** conceptualization, writing – original draft, visualization, supervision, writing – review and editing. **Borbala Blaskovich:** writing – original draft, writing – review and editing.

## Conflicts of Interest

V.S. consulted for and received financial compensation from Roche and Sony and he holds equity in biomentric UG (entrepreneurial company with limited liability, founded in August 2024).

## Data Availability

The data that support the findings of this study are available from the corresponding author upon reasonable request.
